# Dr. Walker, on Dr. Jenner's Diploma

**Published:** 1805-05-01

**Authors:** John Walker

**Affiliations:** Salisbury Square


### *To the Editors of the Medical and Physical Journal.*

Gentlemen,

The discovery, which, as was observed by an intelligent Greek in Egypt, is 'the glory of your isle!' has now made its way through the greater part of the globe; Vacciolation is every where triumphant; the author has had honours showered down upon him by a wondering and grateful world. But, amid the numerous encomiums which have reached your Journal from every quarter, I think ye have not yet been enabled to exhibit a more honourable testimonial than what I have the satisfaction of now presenting to you.

It was handed to me this evening by the Dr. Beku, on his route to Edinburgh, in order to be forwarded to the Discoverer; but as it was our opinion that so signal a mark of approbation should not be locked up from the public by the delicacy of the gentleman to whom it is addressed; I intreat you to publish the whole of this highly flattering Diploma.

The French letter, which accompanied it, I have taken the liberty of translating, and hope you will deem it worthy of the perusal of your Readers.

Your's, respectfully,

*Salisbury Square,* 16, *iv*, 1805. JOHN WALKER.

"To Dr. JENNER,

"*Honourary Member of the Imperial University of Wilna.*

"Sir,

"The Imperial University of Wilna, filled with a profound sentiment of esteem for the immortal Jenner, who has given us the Vaccine, of which the beneficent practice is already spread over the whole surface of the globe, unanimously did itself the gratification of conferring on you, in its sitting of the 13th of November last, the title of Honourary Member. It is with a satisfaction very grateful to me, Sir, that I take the earliest opportunity of communicating the information to you, and of sending you your Diploma.

The Imperial University of Wilna ventures to hope that you will deign to honour it with your correspondence, from which it cannot but promise itself the greatest advantage.

"I have the honour to be, with sentiments of high consideration, "Sir,

"Your very humble and obedient servant,

"J. Stroynowski,

"Bishop Rector of the University of Wilna."

*Wilna, January* 13, 1805.

"P.S. I have the honour, Sir, to recommend to your friendship, Counsellor Beku,* a Professor of our University, who will have the satisfaction to remit to you this letter and your diploma."

---

"Jussu Augustissimi Imperatoris et Authocratoris Totius Rossiae,

"ALEXANDRI I.

"Domini nostri Clementissimi.

"Hyeronymus Stroynowski de Strzemién Nominatus Episcopus Coadjutor Luccoriensis et Zytomiriensis, Praelatus Scholasticus Cathedralis Vilnensis, Eques Ordinis Sancti Stanislai, Theologiae et Juris Utriusque Doctor, Membrum Academiae Scientiarum Florentiae, Academiae Arcadum Romae, Coetus Literarii Varsaviae, et Imperatoriae Societatis Œconomicae Petropoli, Imperatoriae Universitatis

---

* Dr. Beku, Counsellor of His Imperial and Russian Majesty, is Professor of Physiology in the University of Wilna. Ed.

versitatis Vilnensis Rector Magnificus una cum Senatu Academico.

"Cum nuper in consessu literario Vir insigni eruditione et scientia, multisque laboribus conspicuus, accuratissimisque ad tuendam valetudinem praeceptis et observationibus, publicam in lucem editis, potissimum vero perquam nobilis ac primarius et classicus Vaccinae auctor audiat, quo quidem salutaris remedii genere multa mortalium millia praesentissimae morti eripuerit erepturusque sit, non solum de re literaria ac medica, sed de universo genere humano optime meritus EDWARDUS JENNER, Medicinae Doctor unanimi omnium Professorum sententia, in socium Imperatoriae Universitatis Vilnensis sit cooptatus; nos proinde pro munere nostro Eundem Clarissimum et Doctissimum Virum, omnibus juribus ac praerogativis, quae juxta leges Universitatis nostrae, honorificae huic associationi tribuuntur, gaudere testamur. In quorum fidem Diploma hocce manu nostra subscriptum Sigillo Universitatis muniri jussimus. Datum Vilnae in Œdibus Universitatis. XVI. Calendas Decembris, MDCCCIV. anni.

"HYERONYMUS STROYNOWSKI.

"JOSEPHUS MICHIEWIEZ, Decanus Professorum Ordinis Scientiarum Physicarum et Mathemat. Canonicus Cathedralis Samogitiensis. mpp.

"SIMON MALEWSKI, Juris Nat. et Gen. Professor Consiliarius Aul. Secr. Imper. Univer. Vil."

